# Effects of water safety knowledge on high-risk swimming behaviours among Chinese secondary school students: a moderated mediation model

**DOI:** 10.3389/fpsyg.2025.1666979

**Published:** 2025-09-26

**Authors:** Jiaxin Shi, Chao Xie, Hui Zhang

**Affiliations:** School of Physical Education, Hubei Minzu University, Enshi, China

**Keywords:** swimming high-risk behaviours, water safety knowledge, self-efficacy, sensation seeking, junior high school student

## Abstract

**Background:**

The dangers of drowning as a major global public health problem continue to receive international attention. Current literature lacks to describe the mechanism of action of water safety knowledge on high-risk behaviours in swimming. Therefore, the present study constructed a moderated mediation model focusing on the mediating role of sensation seeking in the relationship and the moderating role of self-efficacy.

**Methods:**

In this study, the scale was administered to 1800 junior high school students in central China using the whole cluster sampling method on a school-by-school basis. A total of four scales were measured, which were divided into the Water Safety Knowledge Scale for assessing students’ level of water safety knowledge, the Sensation Seeking Scale for assessing students’ tendency to seek novelty and stimulation, the Self-Efficacy Scale to measure the level of students’ confidence in their own ability to swim, and the Swimming Risky Behaviours Scale to assess the level of students’ knowledge of swimming risky behaviours. Data were analyse and processed using the software AMOS 28.0 and SPSS 27.0.

**Results:**

The results showed that (1) there was a significant negative effect of water safety knowledge on high-risk swimming behaviour after controlling for gender, age, and grade level. (2) There was a significant negative predictive effect of water safety knowledge on sensation seeking, while the positive predictive effect of sensation seeking on swimming high-risk behaviour was equally significant. Sensation seeking mediated the relationship between water safety knowledge and high-risk swimming behaviour. (3) Swimming self-efficacy The direct predictive effect of water safety knowledge on high-risk swimming behaviour and the mediating effect of sensation seeking were both moderated by swimming self-efficacy. For subjects with lower levels of swimming self-efficacy, sensation-seeking had a significant positive predictive effect on high-risk swimming behaviour; for subjects with higher levels of swimming self-efficacy, sensation-seeking had a stronger positive predictive effect on high-risk swimming behaviour.

**Conclusion:**

This study reveals a moderated mediating mechanism whereby sensation seeking influences high-risk swimming behaviours through adolescents’ aquatic safety knowledge, offering significant insights into the complex relationship between safety knowledge and risky behaviours. Based on these findings, it is recommended that schools and relevant authorities move beyond mere knowledge dissemination in future drowning prevention education. Instead, they should develop integrated intervention programs that combine knowledge transfer with self-efficacy enhancement and impulse management training. Tailored, precision-targeted safety education should be implemented for students with different psychological traits (such as high sensation seeking). This approach will more effectively prevent adolescent drowning incidents and promote their physical and mental well-being.

## 1 Presentation of the problem

Globally, at least 236,000 people die from drowning each year, with adolescents being the primary victims (Drowning, 2024). The 2022 China Youth Drowning Prevention Big Data Report reveals that over 50,000 adolescents drown annually in China, and the number of injuries and disabilities far exceeds the death toll (Public Opinion Data Center of People's Daily Online, 2022; CPC, 2015). These alarming figures underscore the urgency and importance of youth drowning prevention education. Research indicates that water safety knowledge is a key factor in preventing drowning and is widely recognized as an effective preventive measure (Xia et al., 2018). Middle school students are in the developmental stage of adolescence, characterized by heightened psychological activity, increased independence, and a strong desire for exploration. However, this unique phase of psychological development also makes them more prone to impulsive risk-taking behaviours (Zhang et al., 2023).

Swimming instruction remains relatively uncommon in China, leaving many students without swimming skills. Even those with some swimming ability often lack adequate water safety knowledge, making it difficult for them to navigate complex aquatic environments and increasing their risk of drowning. In reality, merely possessing swimming skills is insufficient to ensure water safety; students also need sufficient water safety knowledge to accurately assess and manage aquatic risks (Abadi et al., 2024; Moran, 2008). Water safety knowledge plays a crucial dual role in preventing high-risk behaviours. First, it directly guides behavioural choices by influencing information processing. Second, it acts as a moderator that mitigates the impact of other factors potentially leading to high-risk behaviours. Cognitive-behavioural theory posits that individual actions are driven by cognitive and emotional states, with cognitive processes and psychological traits shaping behavioural outcomes. Therefore, incorporating sensation seeking as a mediating variable and self-efficacy as a moderating variable can help elucidate individual differences underlying high-risk swimming behaviour (Xia et al., 2018; Zhang et al., 2017).

Given this, this study will delve into the connection between aquatic safety knowledge and high-risk swimming behaviour, along with its underlying mechanisms, with particular focus on the mediating role of sensation seeking and the moderating effect of self-efficacy. This will help elucidate the pathways through which aquatic safety knowledge influences high-risk swimming behaviour and under what conditions this influence may be weakened. The findings will not only deepen understanding of the relevant influence mechanisms but also provide valuable guidance for helping middle school students correctly apply aquatic safety knowledge and reduce high-risk swimming behaviour.

## 2 Theories and hypotheses

### 2.1 The relationship between water safety knowledge and high-risk swimming behaviour

High-risk swimming behaviour refer to risky behaviours by individuals or groups in open or non-open water environments that are likely to cause harm to their own or others’ health and safety, and their core characteristics include: (1) disregard for water warning signs and safety rules; (2) overestimation of one’s own swimming ability or environmental risk tolerance; and (3) lack of emergency self-rescue skills (Xia et al., 2018). Studies have shown that although water safety knowledge (e.g., risk identification, first aid strategies) shares commonalities with general safety literacy, its uniqueness is reflected in contextualised risk assessment (e.g., current dynamics prediction) and instantaneous decision-dependence, which reinforces the potential of knowledge to directly intervene in high-risk behaviours. Mechanistically, high levels of water safety literacy can reduce risk through a dual pathway: on the one hand, enhancing individual sensitivity to drowning precursors (e.g., offshore currents, physical exhaustion) to reduce overconfidence-induced infractions; and on the other hand, inhibiting impulsive water play decision-making through normative operational memory (e.g., call for help prioritisation, floating techniques) (Abadi et al., 2024; Moran, 2008; Zhang et al., 2017).

Current research on the relationship between knowledge and behaviour based on the Knowledge-Abuse-Behaviour-Pattern (KAP/KABP) is currently being conducted in a wide range of fields, such as drink-driving, consumerism, internet addiction, drug use, smoking, HIV prevention and water safety (Verma et al., 2017; Szucs et al., 2020). The model suggests that individuals acquire knowledge through learning, which is understood and transformed into ideas and concepts, and in doing so, present specific behavioural patterns. In the field of water safety, studies have explored the construction of a water safety education model for primary and secondary school students based on the knowledge, belief, and behaviour model. The study pointed out that among primary and secondary school students, the incidence of high-risk swimming behaviours was significantly lower in the high-knowledge group than in the low-knowledge group, i.e., acquiring more knowledge about swimming safety was thought to contribute to lowering the incidence of high-risk swimming behaviours. This suggests that the level of swimming safety knowledge among primary and secondary school students affects their behaviour when they are active in the waters, which in turn affects the occurrence of water safety accidents (Xia et al., 2018; Zhang et al., 2017). This leads to the following hypothesis: the level of water safety knowledge has a significant negative predictive effect (H1) on an individual’s high-risk behaviour in swimming.

### 2.2 The mediating role of sensation seeking

Sensation seeking is a personality trait in which an individual pursues varied, novel, complex, and intense sensations and experiences, and is willing to take physiological and social risks in order to obtain these experiences (Corr, 1995). Sensation seeking can be influenced by a variety of factors such as an individual’s upbringing, personality traits, and knowledge base (Ravert and Donnellan, 2020). Among them, water safety knowledge, as an important cognitive accumulation of individuals in water-related domains, has a potential moderating effect on sensation seeking. On the one hand, individuals with rich knowledge of water safety can be more aware of the potential dangers in the water environment, thus maintaining a cautious attitude towards water activities that may bring high risks and reducing the tendency of sensation-seeking; on the other hand, individuals with a high degree of sensation-seeking tend to be more inclined to the pursuit of stimulating water activities, ignoring the importance of knowledge of water safety, thus increasing the probability of the occurrence of high-risk behaviours in swimming (Zhang et al., 2023). It has been shown that there is a strong link between sensation seeking and high-risk behaviour in swimming, and high sensation seekers are more likely to participate in challenging and dangerous water activities (Ravindra Singh, 2021). Meanwhile, improved knowledge of water safety can help individuals better assess risks and inhibit impulsive behaviours brought about by sensation seeking (Luo et al., 2024). Cognitive behavioural theory combines the perspectives of cognitive psychology and behaviourism, which suggests that an individual’s behaviour is driven by his/her cognitive and affective states, and that an individual’s cognitive state influences which behaviours he/she adopts. Generally speaking, the amount of cognition can restrain themselves from engaging in risky behaviours, and individuals feel at risk when they act against their cognition and engage in wrong behaviours. In reality, students are often free from cognitive constraints and indulge in risky behaviours because of a momentary ‘feeling of seeking’. Therefore, it is hypothesized that knowledge of water safety may indirectly influence high-risk swimming behaviour through the mediation of sensation-seeking (H2).

### 2.3 The moderating role of swimming self-efficacy

Self-efficacy, proposed by Bandura, refers to an individual’s subjective expectation and judgement of whether he or she can successfully complete a certain behaviour to achieve a desired outcome, and it plays a key role in the behavioural decision-making of an individual facing a complex situation (Bandura, 1977). Knowledge of water safety can enhance an individual’s objective perception of water risk, but if an individual lacks confidence in translating knowledge into behaviour (i.e., low swimming self-efficacy), his or her actual risk avoidance ability may be limited. Research has shown that individuals with low swimming self-efficacy are better at internalising safety knowledge into behavioural strategies that effectively inhibit impulsive sensation seeking (e.g., thrill-seeking swimming behaviours), thereby reducing the risk of high-risk behaviours (Luo et al., 2024). First, from the perspective of cognitive mechanisms, swimming self-efficacy influences individuals’ processing of sensation-seeking information and cognitive judgement of high-risk swimming behaviours. High swimming self-efficacy individuals, with their confidence in their own abilities, are prone to ignore the risks warned by water safety knowledge, driven by sensation seeking. They may be overly concerned with the thrilling experience of sensation-seeking and under-aware of potential dangers, resulting in a greater tendency to fall into high-risk swimming behaviours. On the contrary, individuals with low swimming self-efficacy lack confidence in their ability to cope with the water environment, are able to filter and use water safety knowledge more effectively, and rationally analyse the impulsive information brought about by sensation-seeking, and therefore will be more cautious about sensation-seeking impulses that may trigger high-risk behaviours in swimming (Yu et al., 2024; Benfer et al., 2018). This suggests that swimming self-efficacy may play a moderating role in the relationship between sensation seeking and high-risk behaviour in swimming.

According to Wen and Ye (2014), when the second half of the mediating pathway (i.e., the pathway between sensation-seeking and high-risk behaviour in swimming) is moderated by moderating variables, this mediating effect can be moderated. That is, the extent to which sensation-seeking influences high-risk swimming behaviours will vary at different levels of swimming self-efficacy. For individuals with high swimming self-efficacy, sensation-seeking is more likely to trigger high-risk swimming behaviours because they are less likely to be able to resist sensation-seeking with their ability to use their knowledge of water safety (Zhang et al., 2017; Xia et al., 2018). Secondly, swimming self-efficacy affects individuals’ motivation and goal orientation in swimming activities at the level of behavioural motivation. Individuals with low swimming self-efficacy lack a clear safety goal orientation and sufficient behavioural motivation in swimming activities, and their doubts about their own abilities lead them to develop a strong sense of safety and positive behavioural motivation in the face of sensation-seeking temptations, and they are more inclined to follow the safety rules to ensure their own safety in the swimming process. This strong motivation enables them to better inhibit the disturbance caused by sensation seeking, and even when facing challenging or exciting water situations, they can firmly make correct behavioural decisions based on water safety knowledge to avoid falling into high-risk behaviours (Baretta et al., 2017; McKay et al., 2016). Furthermore, social cognitive theory emphasises the centrality of swimming self-efficacy in individual behavioural decision-making and self-regulation. The theory suggests that swimming self-efficacy influences individuals’ perception of environmental information, expectations of behavioural consequences, and behavioural choices and adherence when faced with potentially risky activities such as swimming (Schunk and DiBenedetto, 2020). Swimming self-efficacy plays a key role in an individual’s ability to self-regulate on the basis of water safety knowledge when influenced by factors such as sensation seeking, which may lead to deviations from safe behaviours, and is an important determinant of whether or not an individual will engage in high-risk behaviours in swimming. Self-efficacy has also been found to play a moderating role in the relationship between an individual’s perception of environmental risk (e.g., knowledge of water safety) and behavioural adaptations (e.g., high-risk behaviour in swimming) (Van Luchene and Delens, 2021). In summary, the present study hypothesised that both the direct predictive effect of water safety knowledge on high-risk swimming behaviour and the mediating effect of sensation seeking would be moderated by swimming self-efficacy (H3).

**Figure 1:**
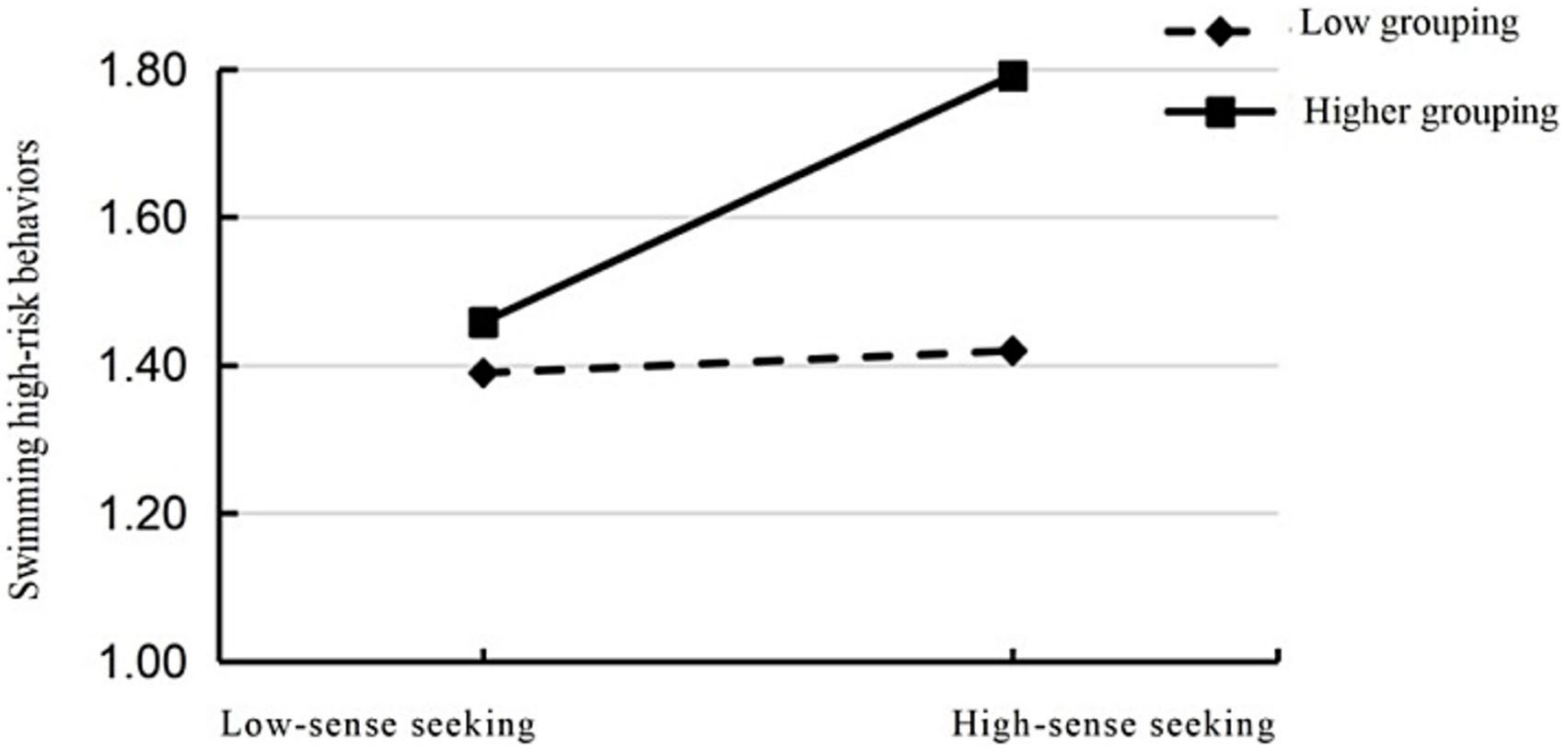
Hypothesised model diagram of the mediating role of sensation seeking and the moderating role of swimming self-efficacy.

A moderated mediation model constructed in this study is shown in Figure 1.

## 3 Research subjects and methods

### 3.1 Subjects

A total of 1,800 students from selected junior high schools in China were tested on the Water Safety Knowledge, Risk Attitude, Self-Efficacy and Swimming Risky Behaviour Scale. The age of the subjects ranged from 13 to 15 years old, of which 863 (51.8%) were boys and 803 (48.2%) were girls; 436 (26.2%) were in the first year, 621 (37.3%) in the second year and 609 (36.6%) in the third year. After the main test explained the instructions in detail, all the subjects completed all the questionnaires in about 45 min, and 1,666 valid questionnaires (92.6%) were collected and collated.

### 3.2 Research tools

#### 3.2.1 Swimming Risky Behaviour Scale

The study employed the Chinese Adolescent Swimming High-Risk Behaviour Scale, which was developed by the research team based on the behavioural and psychological characteristics of Chinese adolescents. Based on the influence factor model constructed through grounded theory in the preliminary phase, combined with relevant literature and expert interviews, a preliminary draft of the Chinese Adolescent Swimming High-Risk Behaviour Pre-test Scale was compiled. After multiple rounds of discussion within the research team and content validity review by five domain experts, the draft was revised for readability, relevance, and logical consistency to form the pre-test version. Following a pre-survey, item analysis, and exploratory factor analysis to refine the items, the scale was administered. After confirmatory factor analysis and reliability/validity testing, the final formal scale was developed. The scale was divided into 4 dimensions belonging to errors, mistakes, general violations and aggressive violations, and included a total of 20 entries. A total of 68.444% of the total variance was explained; the results of the validated factor analysis showed that the scale had a good fit index (χ^2^/df = 3.672, CFI = 0.938, NFI = 0.952, TLI = 0.957, IFI = 0.938, and RMSEA = 0.045); and the total scale had an internal consistency of 0.859, and the dimensional Cronbach ‘s alpha coefficients were all around 0.8, with good scale internal consistency validity. All entries were scored on a Likert 5-point scale, requiring subjects to rate each indicator on a scale of 1–5, with 1 indicating very poor and 5 indicating very good.

#### 3.2.2 Water Safety Knowledge Scale

The Students Water Safety Knowledge, Belief and Behaviour Scale developed by Xia Wen was used to assess the level of students water safety knowledge. The water safety knowledge sub-questionnaire therein was selected as the measurement tool for the study, which contained 10 entries and was rated on a scale of 1–5 (1 = very familiar, 5 = very unfamiliar). The scores of all the entries were summed up to be the total score of the subjects’ water safety knowledge, and the lower the score indicated that the individual had a higher level of water safety knowledge. The Cronbach’s alpha coefficient for the scale was 0.963, indicating good internal consistency of the scale.

#### 3.2.3 Sensation Seeking Scale

The Sensory Seeking Scale revised by Steinberg et al. was used, which consisted of 6 items and was scored on a 6-point scale ranging from 1 point for ‘not at all conforming’ to 6 points for ‘fully conforming’. The average score of all items was taken as the sensation-seeking score, and the higher the score, the higher the level of sensation-seeking. The internal consistency coefficient of the questionnaire was 0.860.

#### 3.2.4 Self-efficacy Scale

The swimming self-efficacy questionnaire developed by Theodorakis was translated to align with the linguistic habits and cultural context of Chinese adolescents. This Chinese version retains all six original items without omission. Participants are asked to rate their level of confidence in completing aquatic tasks of varying difficulty, using a scale ranging from “very confident” (10) to “completely unable” (1), totaling 10 points. The scale demonstrated an internal consistency coefficient of 0.91, with all fit indices meeting psychometric standards. This indicates the scale possesses ideal reliability and construct validity among Chinese adolescents.

## 4 Results

### 4.1 Common method bias test

The common method bias test was carried out using the ‘control of unmeasured single method latent factor method’ recommended by Xiong et al. (2012). The specific operation is as follows: firstly, construct a validated factor analysis model M1, secondly, construct a model M2 containing method factors, and then compare the main fit indices of model M1 and model M2, and the results show that: △χ^2^/df = 0.224, △CFI = 0.003, △TLI = 0.003, △NFI = 0.004, and △RMSEA = 0.001. It can be seen that the common method bias test is based on the ‘controlling for untested single method latent factors’ recommended by Xiong Hongxing et al. 0.001. it can be seen that the magnitude of change of each fitting index is less than 0.01. This indicates that the model fit is not significantly improved after the addition of the common method factor, suggesting that there is no significant common method bias problem in this measurement.

### 4.2 Mean, standard deviation and correlation matrix of each variable

The results of the descriptive and correlation analyses showed (Table 1) that: sensation seeking was significantly positively correlated with swimming self-efficacy and high-risk swimming behaviour, and significantly negatively correlated with knowledge of water safety; swimming self-efficacy was significantly positively correlated with knowledge of water safety and high-risk swimming behaviour; and knowledge of water safety was significantly negatively correlated with high-risk swimming behaviour.

**Table 1:** Results of descriptive statistics, correlation analysis.

Variable	M	SD	Sensation seeking	Swimming self-efficacy	Water safety knowledge	Swimming high risk behaviour
Sensation Seeking	2.764	0.633	1.000			
Swimming Self-Efficacy	2.842	2.244	0.165^**^	1.000		
Water Safety Knowledge	3.554	1.071	−0.203^**^	0.078^**^	1.000	
Swimming High Risk Behaviour	1.511	0.539	0.237^**^	0.276^**^	−0.165^**^	1.000

**p < 0.01.

### 4.3 The effect of water safety knowledge on secondary school students’ high-risk swimming behaviour: a moderated mediating role

Firstly, the mediating effect of sensation seeking in the relationship between knowledge of water safety and high-risk behaviour in swimming was tested by applying Model 4 (which is a simple mediation model) in the SPSS macro prepared by Hayes (2017), controlling for gender, age and grade. The results (see Tables 2, 3 for details) showed that knowledge of water safety was a significant predictor of high-risk swimming behaviour (β = −0.080, t = −6.570, *p* < 0.01). Moreover, when the mediator variable was introduced, the direct predictive effect of water safety knowledge on high-risk swimming behaviour remained significant (β = −0.058, t = −4.809, *p* < 0.01). Meanwhile, there was a significant negative predictive effect of water safety knowledge on sensation seeking (β = −0.119, t = −8.394, *p* < 0.01), while the positive predictive effect of sensation seeking on high-risk swimming behaviour was equally significant (β = 0.180, t = 8.785, *p* < 0.01). In addition, Bootstrap method was used to derive 95% confidence intervals for the direct effect of water safety knowledge on the effect of high-risk swimming behaviours and the mediating effect of sensation-seeking, which did not contain 0 in both the upper and lower bounds (Table 3). This suggests that watershed safety knowledge not only directly predicts high-risk swimming behaviour, but also predicts high-risk swimming behaviour through the mediating effect of sensation seeking. This direct effect (−0.058) accounted for 75% of the total effect (−0.080) and the mediating effect (−0.021) accounted for 25% of the total effect.

**Table 2:** Mediation model test for sensation seeking.

Regression equation (*N* = 1,666)	Fit indicator				Coefficient of significance	
Outcome variables	Predictor variable	R	R2	F(df)	β	t
Swimming high-risk behaviour		0.200	0.040	17.229**		
	Sex				−0.102	−3.894**
	Age				−0.148	−2.930**
	Grade				0.147	2.821**
	Water Safety Knowledge				−0.080	−6.570**
Sensation seeking		0.204	0.042	18.029**		
	Sex				−0.022	−0.725
	Age				−0.014	−0.236
	Grade				0.025	0.411
	Water Safety Knowledge				−0.119	−8.394**
Swimming high-risk behaviour		0.287	0.082	29.851**		
	Sex				−0.098	−3.826**
	Age				−0.146	−2.945**
	Grade				0.143	2.796**
	Sensation seeking				0.180	8.785**
	Water Safety Knowledge				−0.058	−4.809**

The variables in the model are standardised and brought into the regression equation.

**Table 3:** Decomposition of total, direct and mediating effects.

Variable relationship	Efficiency value	Se	Boot CI lower limit	Boot CI upper limit
Total effect	−0.080	0.012	−0.104	−0.056
Direct effect	−0.058	0.012	−0.082	−0.034
Intermediary effect	−0.021	0.006	−0.034	−0.011

Boot’s standard error is the standard error of the indirect effect estimated by the bias-corrected percentile Bootstrap method; the lower Boot CI limit is the value of the lower limit of the 95 per cent confidence interval derived using this method; and the upper Boot CI limit is the value of the upper limit of the 95 per cent confidence interval calculated using this method. All values are rounded to two decimal places.

**Figure 2:**
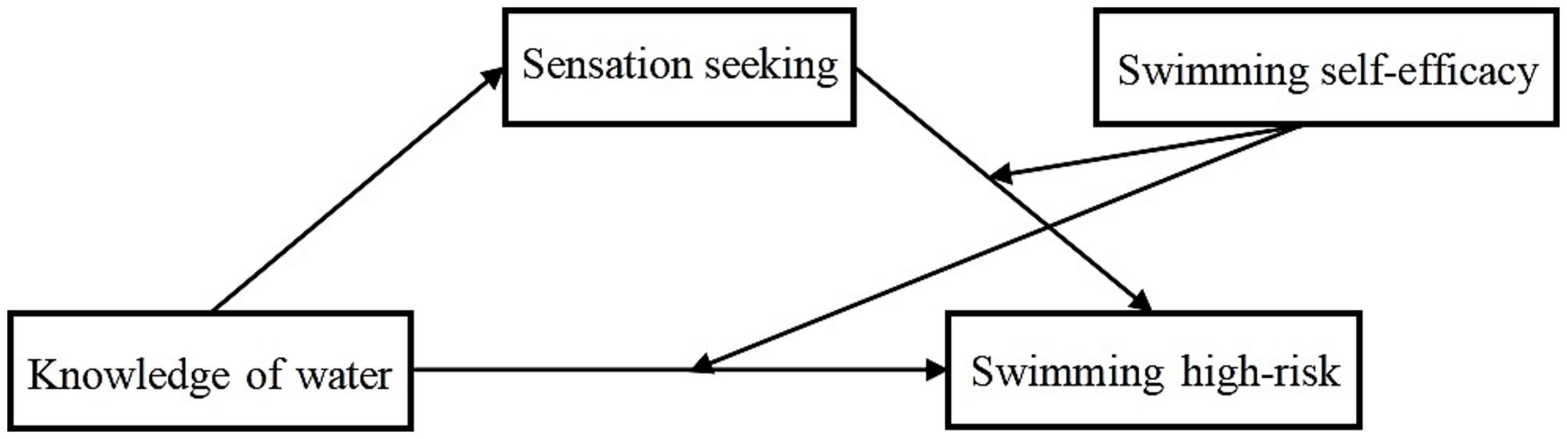
Moderating role of swimming self-efficacy in the relationship between water safety knowledge and high-risk swimming behaviour.

**Figure 3:**
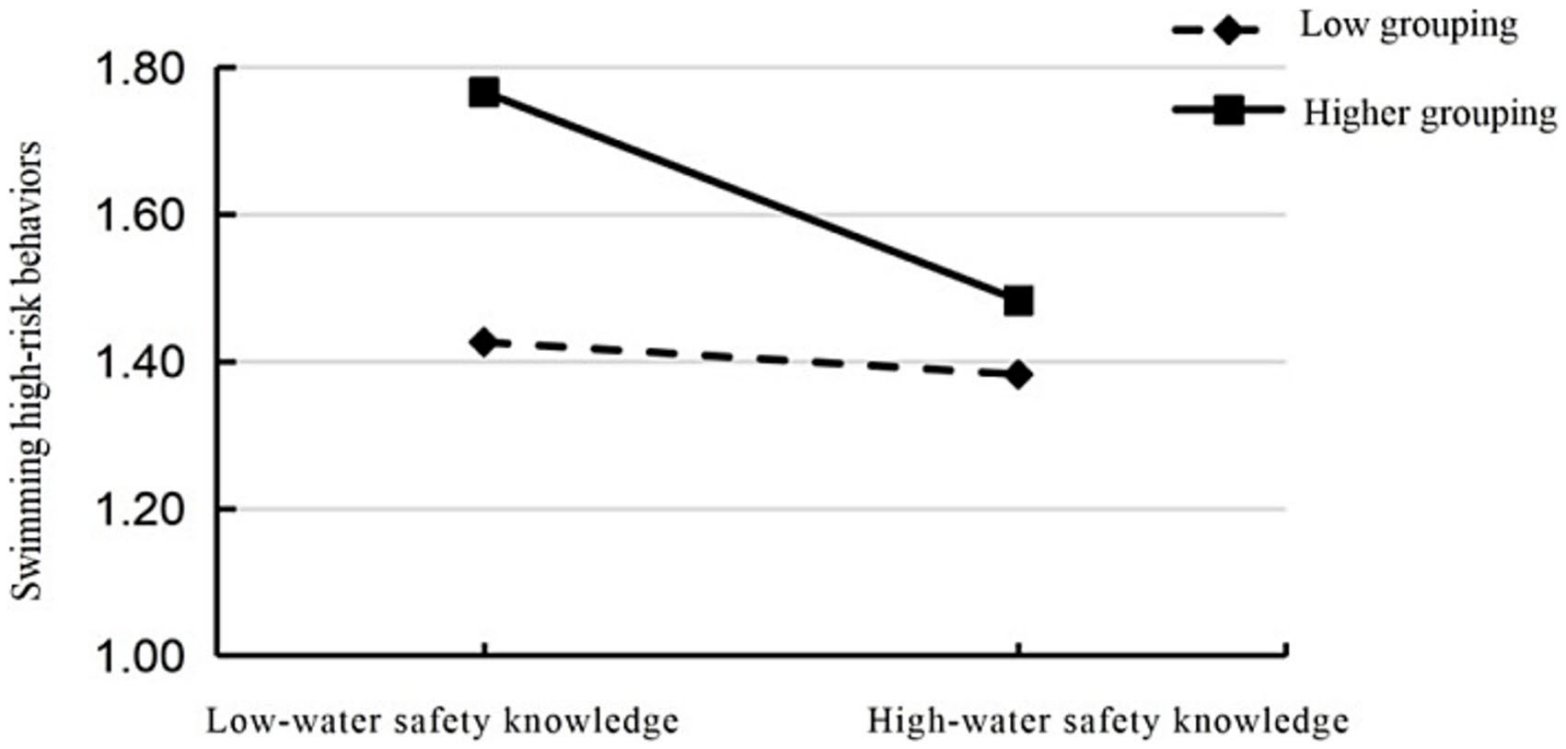
Moderating role of swimming self-efficacy in the relationship between sensation seeking and risky swimming behaviour.

Second, we employed Model 15 from Hayes’ SPSS macro for analysis. During this process, we controlled for variables such as gender, age, and grade level to examine the moderated mediation model. Results (see Tables 4, 5) indicate that after incorporating swimming self-efficacy into the model, both the product of aquatic safety knowledge and swimming self-efficacy, and the product of sensation seeking and swimming self-efficacy significantly predicted high-risk swimming behaviour (high-risk swimming behaviour: (Aquatic safety knowledge × Swimming self-efficacy) β = −0.028, t = −5.874, *p* < 0.01; (sensation seeking × swimming self-efficacy) β = 0.060, t = 8.293, *p* < 0.01). This indicates that swimming self-efficacy not only mediates the direct prediction of aquatic safety knowledge on high-risk swimming behaviour but also moderates the predictive effect of sensation seeking on such behaviours. Further simple slope analysis revealed: As shown in Figure 2, for participants with lower swimming self-efficacy levels (M-1SD), the negative predictive effect of aquatic safety knowledge on high-risk swimming behaviour was not significant: β = −0.016, t = −1.153, *p* = 0.249, 95% unbiased confidence interval [−0.043, 0.011], which included zero. Conversely, for participants with higher swimming self-efficacy (M + 1SD), aquatic safety knowledge exerted a negative predictive effect on high-risk swimming behaviour, and this predictive effect was more pronounced: β = −0.130, t = −8.130, *p* < 0.001, 95% unbiased confidence interval [−0.162, −0.099], excluding zero. Thus, the observed data support the moderating effect of swimming self-efficacy on the direct predictive effect of aquatic safety knowledge on high-risk swimming behaviour in Hypothesis 3, indicating that as individuals’ swimming self-efficacy increases, the protective role of aquatic safety knowledge in reducing high-risk swimming behaviour gradually strengthens (Table 5). As shown in Figure 3, for participants with lower swimming self-efficacy levels (M-1SD), the mediating effect of sensation seeking was not significant: β = 0.021, t = 0.913, *p* = 0.36, 95% unbiased confidence interval [−0.024, 0.066], containing zero. For participants with higher swimming self-efficacy (M + 1SD), sensation seeking exhibited a stronger positive predictive effect on high-risk swimming behaviour: β = 0.265, t = 10.361, *p* < 0.001, 95% unbiased confidence interval [0.215, 0.316], excluding zero. Thus, the observed data support the hypothesis that the mediating effect of sensation seeking in Hypothesis 3 is moderated by swimming self-efficacy. This indicates that as individuals’ swimming self-efficacy increases, the predictive role of sensation seeking for high-risk swimming behaviour also gradually strengthens.

**Table 4:** Mediation model tests with moderation.

Regression equation (*N* = 1,666)	Goodness-of-fit indicator				Significance of coefficients	
Outcome variable	Predictor variable	R	R2	F(df)	β	t
Sensation seeking		0.204	0.042	18.029**		
	Sex				−0.022	−0.725
	Age				−0.014	−0.236
	Grade				0.025	0.411
	Water Safety Knowledge				−0.119	−8.394**
Swimming high-risk behaviour		0.470	0.221	58.641**		
	Sex				−0.062	−2.601**
	Age				−0.189	−4.128**
	Grade				0.171	3.636**
	Sensation Seeking				0.131	6.802**
	Water Safety Knowledge				−0.068	−5.978**
	Swimming Self-Efficacy				0.053	9.570**
	Water Safety Knowledge* Swimming Self-Efficacy				−0.028	−5.874**
	Sensation Seeking* Swimming Self-Efficacy				0.060	8.293**

The variables in the model are standardised and brought into the regression equation.

**Table 5:** Direct and mediating effects at different levels of swimming self-efficacy.

Variable relationship	Swimming self-efficacy	Efficiency value	Se	Boot CI lower limit	Boot CI upper limit
Direct effects	−1.842(M-1SD)	−0.016	0.014	−0.043	0.011
	0(M)	−0.068	0.011	−0.090	−0.045
	2.245(M + 1SD)	−0.130	0.016	−0.162	−0.099
Mediating effects of sensation seeking	−1.842(M-1SD)	−0.01	0.003	−0.008	0.003
	0(M)	−0.02	0.004	−0.025	−0.008
	2.245(M + 1SD)	−0.03	0.009	−0.050	−0.016

In addition, the mediating effect of sensation seeking in the relationship between knowledge of water safety and high-risk swimming behaviours tended to decrease at all three levels of swimming self-efficacy (Table 5). That is, as the level of subjects‘swimming self-efficacy increased, water safety knowledge was more likely to trigger students’ high-risk swimming behaviour by enhancing their sensation seeking.

## 5 Discussion

In view of the growing concern about the life safety hazards of adolescents’ high-risk swimming behaviours, there has been a rapid growth in related research in recent years. Existing studies have mostly focused on the influence of swimming skill level, but neglected the predictive role of multiple factors such as water safety knowledge. This single perspective not only simplifies the complex psychological mechanism of behavioural decision-making, but also makes it difficult to systematically reveal the core proposition of ‘what characteristics of individuals lead to behavioural differentiation due to differences in water safety knowledge in specific situations’. Based on the theory of knowing, believing and acting, this study innovatively constructs a mediation model of water safety knowledge. The breakthroughs of this study are: first, it shifts the research perspective from the traditional skill dimension to the cognitive decision-making process, which opens up a new path for drowning prevention research; second, it provides double theoretical support for the construction of a precise water safety education system by revealing the chain transmission mechanism of ‘knowledge transmission-mental drive-behavioural output’ and its boundary conditions. By revealing the chain transmission mechanism of ‘knowledge transmission—psychological drive—behavioural output’ and its boundary conditions, it provides dual theoretical support for the construction of a precise water safety education system.

### 5.1 Relationship between water safety knowledge and risky swimming behaviour

This study found that aquatic safety knowledge exhibits a significant negative correlation with high-risk swimming behaviour and directly predicts such behaviours negatively. This validates Hypothesis 1 and aligns with the core tenets of cognitive behavioural theory: an individual’s cognition (knowledge) serves as a critical prerequisite influencing behavioural decisions (Bandura, 1977). The findings further confirm that enhancing aquatic safety knowledge represents a key cognitive intervention strategy for preventing high-risk swimming behaviour among students.

The complexity of this study’s model suggests that the “knowledge–behaviour” relationship may not follow a simple linear process. While knowledge holds particular importance during adolescence, the social environment (such as peer pressure) and individual psychological traits may either weaken or enhance its efficacy. This study incorporates sensation seeking and swimming self-efficacy to explore the specific conditions and mechanisms through which knowledge becomes effective. Previous research indicates that comprehensive safety knowledge can effectively reduce risky behaviours across various scenarios. In swimming contexts, understanding aquatic safety knowledge helps adolescents identify potential risks, such as the characteristics of different water bodies and the danger levels of currents (Morrongiello et al., 2010). Although multiple factors contribute to high-risk swimming behaviour, aquatic safety knowledge can be considered a crucial factor in preventing such behaviours among adolescents. When students possess sufficient aquatic safety knowledge, they become more aware of dangerous behaviours, thereby reducing the likelihood of swimming in hazardous waters. Furthermore, when faced with inappropriate peer invitations or temptations toward risky behaviours, they are better equipped to make sound judgments and refuse such invitations. The junior high school years represent a critical developmental stage for adolescents, during which drowning accidents have become a major threat to their safety. This underscores the urgent need for aquatic safety education—a systemic endeavor involving multiple levels including society, schools, and families. It requires collaborative efforts from all parties, focusing not only on imparting knowledge but also on helping adolescents with diverse psychological traits apply this knowledge in practice (Xia et al., 2018; Zhang et al., 2023). Only through comprehensive, in-depth, and sustained water safety education can adolescents’ awareness of water safety and self-protection capabilities be effectively enhanced, thereby reducing the occurrence of drowning incidents.

### 5.2 The mediating role of sensation seeking

Sensory seeking, a stable personality trait characterised by an individual’s pursuit of novelty, complexity, and adventure, has been extensively demonstrated as a consistent predictor of various risky behaviours among adolescents (Mehpare Tokay et al., 2023; Nesayan et al., 2018). This study provides the first validation within the aquatic safety domain that sensory seeking serves as a key psychological mediating mechanism linking aquatic safety knowledge to high-risk swimming behaviours.

The study reveals that, within the context of secondary school students’ swimming behaviours, insufficient aquatic safety knowledge may constitute a cognitive gap that prompts high sensation seekers to engage in risky activities. This aligns with the ‘stimulus-value’ decision-making model: when individuals lack cognitive awareness of the value of potential dangers (safety knowledge), the intrinsic drive for sensory stimulation (sensation seeking) becomes dominant, thereby increasing the occurrence of high-risk behaviours (Chunmei et al., 2021; Zhang et al., 2023). Furthermore, the impulsivity often accompanying high sensation seeking may cause individuals to suppress acquired safety knowledge in specific contexts (e.g., peer pressure), instead pursuing immediate stimulation (Dodig Hundric et al., 2023; Leavy et al., 2022). Thus, this study’s mediational model reveals a clear pathway: adequate aquatic safety knowledge helps inhibit sensation-seeking tendencies, thereby reducing high-risk swimming behaviours driven by stimulation-seeking. This suggests that interventions may prove more effective if they shift focus from mere risk avoidance towards providing high sensation seekers with alternative, safe stimulating activities (such as competitive swimming or diving under supervision), while reinforcing their ability to recall and apply safety knowledge in impulsive situations.

### 5.3 The moderating role of self-efficacy

This study validated the complex dual moderating role of swimming self-efficacy within the model. Findings revealed that higher swimming self-efficacy amplified the negative impact of inadequate aquatic safety knowledge on high-risk swimming behaviours.

Specifically, for individuals with high swimming self-efficacy, the mediating pathway through which aquatic safety knowledge influences high-risk swimming behaviours via sensation seeking was more pronounced. This discovery can be explained by the ‘overconfidence effect’ within cognitive behavioural theory. Individuals with high self-efficacy may overestimate their swimming abilities, thereby underestimating real-world risks and becoming more inclined to translate sensation-seeking into actual high-risk behaviours (Luo et al., 2019,). In contrast, those with low self-efficacy exhibit more cautious behavioural patterns, where the protective effect of safety knowledge exists but is less pronounced. This finding aligns strongly with self-regulation theory (Bandura, 1997). High self-efficacy individuals may experience biases in self-regulation: while confident in their abilities, this confidence may diminish their emphasis on safety knowledge (Spooner et al., 2024). Consequently, when confronted with stimulus-seeking impulses, their self-regulatory mechanisms may prove ineffective (Myriam Guzman et al., 2024; Trautner and Schwinger, 2020) Conversely, low self-efficacy individuals, through their conservative assessment of their capabilities, maintain heightened vigilance, thereby partially compensating for deficiencies in safety knowledge. Previous research indicates that high self-efficacy may, in certain contexts, lead to unintended increases in risk-taking behaviour, particularly when individuals possess inadequate risk perception (Siraj et al., 2021; Thurm et al., 2022). The present study’s findings further validate the applicability of this conclusion within the aquatic safety domain.

From a practical perspective, merely enhancing swimming self-efficacy without concurrently strengthening risk awareness and education may prove counterproductive. Effective interventions require balanced development of both self-efficacy and safety knowledge to prevent increased risk-taking stemming from overconfidence. Particularly for high self-efficacy individuals, it is essential to reinforce risk perception education, thereby fostering more comprehensive and accurate risk assessment capabilities.

### 5.4 Research limitations

Finally, this study has certain limitations. All data are based on self-reporting and may be subject to bias; the sample comprises Chinese secondary school pupils, necessitating further validation of the findings’ cultural generalisability. These limitations point to directions for future research, such as longitudinal studies of peer influence, incorporating objective behavioural measures, and testing this model across different cultural contexts.

## 6 Conclusion

1. There is a significant negative effect of water safety knowledge on high-risk swimming behaviour after controlling for gender, age, and grade level.
2. There was a significant negative predictive effect of water safety knowledge on sensation seeking, while the positive predictive effect of sensation seeking on high-risk behaviour in swimming was equally significant. Sensation seeking mediated the relationship between water safety knowledge and high-risk swimming behaviour.
3. Swimming self-efficacy The direct predictive effect of water safety knowledge and the mediating effect of sensation-seeking on high-risk swimming behaviour were moderated by swimming self-efficacy. For subjects with lower levels of swimming self-efficacy, sensation-seeking had a significant positive predictive effect on high-risk swimming behaviour; for subjects with higher levels of swimming self-efficacy, sensation-seeking had a stronger positive predictive effect on high-risk swimming behaviour.
4. Based on the research findings, we recommend that schools and relevant departments should not limit future drowning prevention education to knowledge dissemination alone. Instead, they should develop integrated intervention programs that combine knowledge transfer with self-efficacy enhancement and impulse management training. Furthermore, differentiated and targeted safety education should be implemented for students with distinct psychological traits (such as high sensation seeking). This approach will more effectively prevent adolescent drowning incidents and promote their physical and mental well-being.

## Data Availability

The raw data supporting the conclusions of this article will be made available by the authors, without undue reservation.
